# Pharmacological and Phytochemical Appraisal of Selected Medicinal Plants from Jordan with Claimed Antidiabetic Activities

**DOI:** 10.3797/scipharm.1212-20

**Published:** 2013-10-15

**Authors:** Fatma U. Afifi, Violet Kasabri

**Affiliations:** Faculty of Pharmacy, The University of Jordan, Queen Rania Al-Abdullah Street, 11942 Amman, Jordan.

**Keywords:** Traditional medicine, Medicinal plants, Diabetes, Jordan, Ethnomedicine

## Abstract

Plant species have long been regarded as possessing the principal ingredients used in widely disseminated ethnomedical practices. Different surveys showed that medicinal plant species used by the inhabitants of Jordan for the traditional treatment of diabetes are inadequately screened for their therapeutic/preventive potential and phytochemical findings. In this review, traditional herbal medicine pursued indigenously with its methods of preparation and its active constituents are listed. Studies of random screening for selective antidiabetic bioactivity and plausible mechanisms of action of local species, domesticated greens, or wild plants are briefly discussed. Recommended future directives incurring the design and conduct of comprehensive trials are pointed out to validate the usefulness of these active plants or bioactive secondary metabolites either alone or in combination with existing conventional therapies.

## Introduction

Diabetes mellitus (DM) is highly recognised as the most common metabolic and endocrine disorder worldwide. It is linked to disturbances in carbohydrate, fat, and protein metabolism [1]. It is especially important because the global prevalence of diabetes is projected to escalate relentlessly. At least 250 million individuals worldwide suffer from diabetes and this number will double by 2030. Increases in complications will undeniably follow increasing diabetes incidence rates [2]. More than 80% of diabetes deaths take place in low- and middle-income countries [3].

The regional prevalence of diabetes in MENA (Middle Eastern and North Africa) countries is 7.7%. Locally, endocrine, nutritional, and metabolic diseases represent 7.9% of deaths in Jordan [3–5]. With a prevalence rate at 10.1%, Jordan has the ninth highest incidence of diabetes among neighbouring countries. Several national surveys designated that the prevalence of type 2 diabetes and impaired fasting glycemia is unprecedentedly high, amounting to an epidemiological transition in Jordan [6–8].

**Fig. 1:**
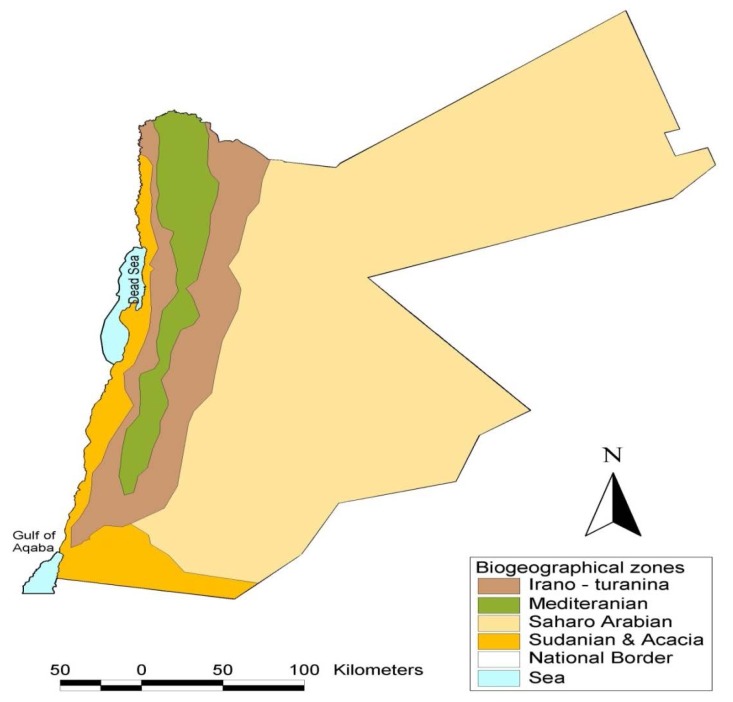
Biogeographic zones of Jordan

Undoubtedly, Jordan’s habitat is exceptional. It is at the intersection of arid desert, dense forest, and tropical geography, thus bestowing the country with a rich variety of plants and microorganisms that can be resourcefully studied (Fig. 1) [9]. The heterogeneous ecological conditions have favoured the proliferation of more than 2,500 wild plant species from 700 genera; of these, there are approximately 100 endemic species, 250 rare species, and 125 very rare species [9–11]. Unfortunately, this substantial biodiversity is principally understudied, or even worse, left unexplored [9–12].

Apparently, there is a repository of ethnobotanical studies in the Mediterranean basin, providing a new and key tool for a quest after invaluable phytopharmaceuticals or the development of functional foods or nutraceuticals [13–20]. Traditional medicine practices, being part of the Jordanian culture, are considered responsible for an impartial role in primary health care despite modern medicine accessibility [21] where vegetables, culinary herbs, and medicinal plants are among the main choices in the management of diabetes [13, 21–30]. Essentially important, traditional medicine has not only survived, but thrived in the transcultural environment and intermixture of many ethnic traditions and beliefs despite the ‘aging’ or ‘vanishing’ of folk phytotherapy in the sense that the wealth of knowledge of medicinal plants resides mostly in elderly rural people with modest tuition [31]. Also, it is officially neither integrated in the health care system nor recognized in the national policies of the country. Furthermore, as the use of medicinal plant remedies constitutes the common legacy of Jordanians, reliability fractions on herbal medicine vary from rural and desert areas to heavily populated urban ones [21–24]. In the last decades, more plants have been added to the list of endangered species. This results in the urgent inevitability for local communities to implement nationwide conservation and sustainability programs [32].

The objective of this review is to emphasize the ethnopharmacological practices related to 20 selected ethnobotanicals with claimed antidiabetic properties in light of their comprehensive scientific evaluation and bioactive plant secondary metabolites. Considering the hugely diverse plant species in diabetes traditional medicine, the present manuscript can be complementary to our previous report of 30 indigenous plants [33]. In fact, all our attempts in this direction serve to bring together the Jordanian inventory of diabetes ethnomedicine. Still, further studies might also be integrated into this line of work.

## Results and Discussion

Based on centuries of beliefs and observations, plants are primarily used in preparatory forms of infusions or decoctions in ethnomedicinal practices. Worldwide, more than 1,200 species of plants have been reported to be used empirically for their claimed antidiabetic activity [34] while in the Jordanian traditional medicine, almost 70 plant species are used by diabetic patients. Although indigenously grown plants are consumed in the countryside, in the vast cities, including the capital Amman, however, the herbalists’ shops display a wide variety of imported plant species, like *Zingiber officinalis*, *Terminalia chebula,* or *Emblica officinalis*, alongside the likely obtainable native ones [11, 23, 35, 36].

On the other hand, reports on the concomitant use of plants in orthodox therapy are evidently understated. In this aspect, interviews with diabetes patients in specialized health centres in Jordan further signified a more diversified list of selected plants [21, 26]. The reported plants were: *Camellia sinensis, Pimpinella anisum, Zingiber officinale, Matricaria recutita, Salvia fruticosa, Trigonella foenum-graecum, Nigella sativa, Lupinus albus, Teucrium polium, Allium sativum, Cinnamomum zeylanicum,* and *Olea europea.* It is tempting to speculate that the high frequency of use is related to the high efficacy and safety of the plant material, such as green tea, aniseed, or chamomile, although there are no clinical studies to indicate monitoring of glucose and haemoglobin A1c levels in diabetic patients using these plants [31]. Also, there is no information available on the protection from target organ damage by the long-term use of plant products. Interestingly, white lupin (*Lupinus albus)*, fenugreek (*Trigonella foenum-graecum)*, garlic (*Allium sativum*), olive leaves (*Olea europea)*, ginger (*Zingiber officinale)*, felty germander (*Teucrium polium*), or black fennel (*Nigella sativa)* were not the top/main preference herbs of choice by the Jordanian diabetic patients [21, 26], despite being scientifically appraised for their antidiabetic activities and frequent use in communities abroad. This has lent further weight to our major interests and concerns stemming from the unjustified claims and selection pressure of certain herbal ethnomedicines in the treatment of diabetes.

Obviously, the significant efficacy of hypoglycaemic herbs, obtainable, via functioning as pancreatic insulin secretagogues and extrapancreatic insulin mimetics, enhancing glucose uptake by adipose and muscle tissues, or via inhibiting hepatic gluconeogenesis and intestinal carbohydrate digestibility and absorption, is comparable to conventional diabetes pharmacotherapeutics [37–39]. Literature surveys of botanicals with traditional uses, critically withstanding pharmacological appraisal, indicated that local target-based and mechanistic reports on diabetes interventional phytotherapies are primarily limited and inadequate. Gharaibeh et al. [40] investigated the hypoglycaemic effects of the aqueous extract of *Teucrium polium* in normal and streptozocin (STZ)-diabetic rats. Additionally, the hypoglycaemic effects of *Ballota nigra*[41] and *Artemisia sieberi*[42] were evidenced in alloxan-diabetic rats. Also, the antioxidative properties of an extensive list of Jordanian plants with diabetes ethnotherapeutic claims were closely discussed [43]. In other studies from Jordan, the pancreatic effects of the antidiabetic plants *Eriobotrya japonica*[44] and *Ferula asafoetida* were reported [45]. Further comprehensive *in vitro* and *in vivo* examinations of indigenous herbs valued as antidiabetic phytomedicines, including *Achillea santolina*, *Eryngium creticum*, *Geranium graveolens, Paronychia argentea, Pistacia atlantica, Rheum ribes, Sarcopoterium spinosum*, *Teucrium polium,* and *Varthemia iphionoides,* have been recognised with elaboration [46–49]. These research findings could collectively resonate with the prevention/modulation of postprandial hyperglycaemia, budding from the natural therapeutic inhibitors of α-amylase and α-glucosidase, with ethnopharmacological claims in the local communities.

Table 1, demonstrating the antidiabetic and/or other pharmacological activities of the compiled 20 plants, provides an updated overview of their reported phytoconstituents as well. In the present review, flavonoids are among the major classes of secondary metabolites detected in most of the tabulated plants. The antidiabetic activity is well-documented for numerous flavonoids [50]. *Achillea santolina* and *A. fragrantissima* are widely distributed in Jordan and used for their claimed antidiabetic activities. In STZ diabetic rats, hypoglycaemic activity was only evaluated for the former species though both species are rich in flavonoids among other similar volatile oil constituents. Hence, an antidiabetic activity can be likely assumed and verified for flavonoid-rich *A. fragrantissima*[51]. Also, the promoted antidiabetic activity of *Anthemis pseudocotula* might be due largely to its flavonoid content. On equal footing, similar postulations can be deduced for plant species with reported antioxidative capacities. Basically, natural antioxidants are well-linked with antidiabetic therapeutic/preventive pharmacology [34, 43, 52–55]. Consequently, despite the lack of scientific scrutiny, it can be speculated that the antioxidative propensities of *Alhagi marourum, Alchemilla vulgaris*, *Cucurbita maxima*, *Juniperus phoenicea, Quercus coccifera,* and *Ambrosia maritima* can in principle justify their reported phytotherapeutic claims and ethnomedicinal uses.

Six of the enlisted plants, namely *Ajuga iva*, *Cleoma droserifolia*, *Urtica dioica, Sarco-poterium spinosum*, *Rheum ribes, Zea mays,* and *Geranium graveolens* exhibited hypoglycemic activity in STZ and/ or alloxan diabetic animal models via inhibition of α-amylase and/or α-glucosidase or glucose absorption as plausible *in vitro* action mechanisms among many others (Table 1). On the other hand, neither *in vivo* nor *in vitro* bioactivity could be detected in antidiabetes pharmacology appraisals with *Peganum harmala* or *Ferula persica.* These findings strongly negate the claimed ethnotherapeutic uses promoted for these plant species. As for *Varthemia iphionoides* and *Zizyphus spina-christi*, the lack of complementary *in vivo* or *in vitro* testing necessitates further experimental design and verification on future accounts [56].

The hypoglycaemic properties of several classes of phytochemicals, including alkaloids, flavonoids, glycosides, glycolipids, polysaccharides, peptidoglycans, carbohydrates, amino acids, saponins, and terpenoids, have been exhaustively reported in the literature [37, 38, 57–60]. Additionally, it is well-accepted that certain herbs may alleviate considerably evident hyperglycaemia in clinical trials with well-characterised mechanisms of action [61, 62]; their test results, however, are subject to multiple factors. Among which, different parts of an herb may have different ingredient profiles or different extraction methodologies may yield diverse active ingredients. In addition, each plant species contains multiple compounds, only a few of which may be therapeutically effective either alone or acting in synergism [63, 64]. Hence, an urgent need exists for research proceedings in identifying the phytoconstituent(s) directly associated with hypoglycaemic/antihyperglycemic bioactivity with equivalent assessments of the intra- and inter-species variations in secondary metabolites. Future research directives may also incur extensive clinical population-based studies for selected species. Moreover, investigating the combination formulations of natural products with synthetic drugs of complementary pharmacologies may determine the optimal and cost-effective therapies. Additionally, as herb-drug interactions in diabetic treatments/supplements have not been well-evidenced or documented [65], it is warranted that follow-up studies on their long-term side-effects be conducted. Subsequently, this may invite the potential development of food products fortified with clinically safe and effective plant extracts and possible downstream planning and incorporation into diabetic diets [66].

In conclusion, the reported findings, uniquely indicating the potential use of medicinal plants as antidiabetic agents, are among the very few that explored Jordanian flora from semi-arid and arid bioclimatic areas for pharmaceutical leads. Comprehensive research aiming at fully exploiting any of the promising species from the Jordanian flora, either alone or in combination with existing therapies, might lead to discovery of new avenues for medicinal plants/natural compounds in reducing the major public health impact of diabetes. Characterization of molecular targets and elucidation of relevant mechanisms of action also stand for another set of plausible requirements. Then, despite modern medicine accessibility, traditional medicine can be propagated as a viable health alternative.

## Figures and Tables

**Tab. 1:** Antidiabetic plants indigenous to Jordan used for the treatment of diabetes in folk medicine in Jordan.

No	Species	Reported antidiabetic efficacy and/or mechanism of action	Other reported pharmacological effects
	Reported phytoconstituents		
1	Asteraceae *Achillea fragrantissima* (Forsk.) Sch. Bip (Infusion of leaves and shoots [23]) Flavonoids [67–69]. Essential oil (santolina alcohol, artemisia alcohol, artemisia ketone, *cis*-thujone and *trans*-thujone, 1,8-cineole, fragranol, fragranyl acetate and terpin-4-ol) [70].	NONE	Antioxidative effects [43]. Lacked any antirheumatic or anti-inflammatory effects in carrageenan-induced acute inflammation in rats [71], but exerted antimicrobial and antiviral activities [70, 72–75]. Modulatory effects on rat ileum muscle contraction [74]. Beneficial in preventing/treating neuro-degenerative diseases [76]. Aqueous extract exhibited strong cytotoxicity and larvicidal activities [77, 78].
2	Asteraceae *Achillea santolina* L. (Infusion of leaves, flowering branches [24]) Flavonoids such as luteolin, quercetin, cosmosiin, hyperoside and cynaroside [79–81], terpenoids [82]. Essential oil (1,8-cineole, fragranol, fragranyl acetate and terpin-4-ol) [70].	Hypoglycemic activity in STZ rats due to antioxidative potential [51, 83–84]. Lack of significant inhibition of α-amylase and α-glucosidase *in vitro* despite acute antihyperglycemic trend in starch fed rats [48].	Enhancement of antimicrobial efficacy against antibiotic resistant *E. coli* and other microorganisms [85, 86]. Potent anti-inflammatory and immunomodulatory activities [87].
3	Asteraceae *Ambrosia maritima* L. (Infusion of herb [24]) Sesquiterpenes and sesquiterpene lactones [88–90]. Thiophene A and thiophene A diol as major polyacetylenes [91].	NONE	Cytotoxicity [88]. Effective molluscicidal activity [92–96] but little or no effect on the larvae of *Anopheles stephensi* and *Aedes aegypti*[97, 98] as well as hepatoprotective and antioxidant properties [99]. Antifungal activity of its sesquiterpenes [89].
4	Asteraceae *Anthemis pseudocotula* Boiss (Infusion of flowering heads, leaves [24]) Flavonoids (apigenin, apigenin-7-glucoside) and coumarins (scopoletin and herniarin) [100]. Essential oil [101], sesquiterpenes and sesquiterpene lactones [102, 103].	NONE	NONE
5	Asteraceae *Varthemia iphionoides* Boiss and Blanche (Decoction of shoots, leaves [23, 25]) Eudesmane sesquiterpene [104]. Flavonoids: jaceidine, kumatakenine, xanthomicrol, seven 3-methoxyflavones [105–107]. Essential oil [108, 109].	Inhibitory activity against porcine pancreas α-amylase [110]. Highly significant dose dependent dual anti-α-amylase and anti-α-glucosidase efficacies *in vitro*[49]. Significant decreases in the blood glucose levels of the STZ hyperglycaemic rats and hypoglycaemic activity in the diabetic sand rats [111, 112].	Antiplatelets benefits [113] as well as antioxidative effects [43, 105, 110, 114]. Cytotoxic effect on human leukemia (HL-60) and antitumor properties [105, 115]. Pronounced antibacterial and antifungal propensities [86, 105, 106, 116].
6	Capparaceae *Cleoma droserifolia* (Forskal) Delil (Decoction of leaves [24]) Terpenes, flavonoids (quercetin, kaempferol, and isorhamnetin) and phenolic acids [117–122].	Hypoglycaemic efficacy via potentiation of peripheral and hepatic insulin sensitivity, thus decreasing hepatic glucose output. Also decreasing intestinal glucose absorption, which was evident by blunting plasma glucose levels throughout the oral glucose challenge in tetracycline-induced fatty liver rats [123]. Insulin induction activity [124]; restored the blood glucose level, plasma malondialdehyde, and urine sugar to near the physiological values [121]. In alloxan-induced diabetic mice reduced oxidative stress in addition to antihyperglcemic activity [125].	Suppressive effect on NO production in activated macrophages *in vitro*[117]. Hepato-protective effect [119]. Hypocholesterolemic and protective anti-atherogenic benefits in tetracycline induced fatty liver in rats [123]. Hypolipidemic, antioxidative and anti-*Schistosomiasis mansoni* properties [124–126]. Hepatotoxicity in co-culture systems [127]. Significant cytotoxic activity against breast (MCF7) and colon (HCT116) cancer cell lines [122].
7	Cucurbitaceae *Cucurbita maxima* Duchesne (Dry seeds [23]) Spinasterol [128]. Carotenoids (violaxanthin, beta-carotene) and lutein [129]. Tocopherols, fatty acids (oleic, linoleic, and palmitic acids), beta sitosterol and phenolic acids [130–133]. Water soluble polysaccharide fraction [134]. Volatile compounds, such as lipid aldehydes, ethyl acetate, 2,3-butanedione, and dimethylsulfide [135].	Wistar rats treated for 70 days with pumpkin seed flour exhibited significant decrease in glucose and triacylglycerides [136].	Antigenotoxic principle [128] and antioxidative benefits [134]. Trypsin inhibition [137, 138]. Larvicidal, ovicidal and repellent properties against mosquito bites [139].
8	Cupressaceae *Juniperus phoenicea* L. (Decoction of fruits, leaves [13]) Lignans [140]. Phenylpropane glycosides [141], essential oil (α-pinene, α-and β-phellandrenes, α-terpinyl acetate, Δ^3^ carene and myrcene) [142–156]. Oxygenated diterpenes [157]. Terpenic hydrocarbon fraction dominance [158–160]. Polyphenols, flavonoids and essential oil from the fleshy cones [161–164]	NONE	Anticancer constituents [140] and cytotoxicity against 5 cell lines [156, 157]. Antimicrobial properties and helpful in the prevention of aflatoxin contamination for many foods [144, 150, 153, 154, 156, 159, 160, 163–166]. Potent activity against *Candida albicans*[143]. Antiparasitic, nematicidal and antifouling constituents [155, 167] with tick repellent properties [168]. Antioxidative [152, 159, 160, 162, 164, 166] propensities. Remarkable effect in enhancing liver and kidney functions in CCl_4_ treated rats, and may thus be of therapeutic potential in treatment of hepatotoxicity and nephrotoxicity [169, 170]. Wound-healing effect [171]. Anticholinesterase activity [148, 166].
9	Fagaceae *Quercus coccifera* L. (Decoction of galls [13]) Polyphenols and tannins (pedunculagin, castalagin, phillyraeoidin A, and acutissimin B) [164, 172, 173]. Sesquiterpenes [174].	NONE	Antioxidant and antibacterial properties [164]. Anti-lipoperoxidant properties-related gastroprotective and anti-ulcerogenic effects [173, 175]. Anthelmintic activity against parasitic nematodes [176].
10	Geraniaceae *Geranium graveolens* L. (Decoction of leaves [13, 24]) Essential oils [177–182].	Dual inhibition of α-amylase and α-glucosidase *in vitro*, confirmed by highly significant and potent acute antihyperglycemic trends in starch-fed rats [49].	Fumigant antitermitic activity [179]. Antioxidant activity [182]. Repellent effect against host-seeking nymphs of *Ixodes ricinus*[183] with antimicrobial qualities [180, 184, 185]. Mosquito repellent property [186]. Improves the immune cell count of cancer patients receiving chemotherapy and/or radiotherapy to prevent leucopenia and immune impairment that usually occurs during cancer therapy [187].
11	Labiatae *Ajuga iva* L. (Schreber) (Decoction of herb [24]) 14,15-dihydroajugapitin [188] Ecdysones [189] and phytoecdysteroids [190, 191]. Iridoids, such as 8-*O*-acetylharpagide [192, 193].	Its phytoecdysteroids are beneficial for correcting the hyperglycaemia and preventing diabetic complications in liver, pancreas and kidneys in alloxan diabetic rats [191]. Acute and subchronic antihyperglycemic effects in normoglycemic and STZ-diabetic rats [194, 195].	Hypolipidemic and hypocholesterolemic activities that may reduce intestinal cholesterol absorption [195–200] as well as antiatherogenic efficacy [199]. Vasorelaxant effect in rat aorta [196]. Reducing the oxidative stress in hyper-cholesterolemic rats by increasing the antioxidant enzymes activity [200]. Antioxidative benefits [201]. Inhibits crystallization of calcium oxalate in the urine [202]. Insecticidal properties [203, 204].
12	Leguminoseae *Alhagi maurorum* Medicus (Decoction of roots [24]) Flavonoids (isorhamnetin-3-*O*-[-α-1-rhamnopyranosyl-(1→3)]-β-D-glucopyranoside; 3′-*O*-methylorobol and quercetin 3-*O*-β-D-glucopyranoside) [205, 206]; cinnamic acids, phenolic acids, β-sitosterol and its glucoside [205, 207]. Three flavones (2-phenyl-1,4-benzopyrone derivatives) [208]. Polymethoxy substituted flavanenol [209] and triterpenoid lupeol [210]. Tannins and anthraquinones [211].	NONE	Antioxidative [206, 207, 212], anti-inflammatory [208, 210, 213, 214], antifungal [211] and anti-gastric ulcer [208, 214–216] activities. Antinociceptive [217] and antidiarrhoeal effects [218]. Spasmolytic and urether relaxing benefits [209, 219, 220]. ACE- and NADH oxidase-inhibitory activity [221]. Antibacterial activity [222]. Potent allelopathic activity [223].
13	Poaceae *Zea mays* L. (Decoction of kernel [26]) Feruloylated oligosaccharide [224]. Flavone C-glycosides and sesquiterpenes [225, 226]. Phenolics (proto-catechuic acid mainly) [227]. Hydroxycinnamic acids [228]. Anthocyanins (cyanidin 3-glucoside and cyanidin-3-(6″-Qmalonylglucoside) [229].	*In vitro* inhibition of glycation [225]. Suppressed the progression of diabetic glomerular sclerosis in STZ- diabetic rat [230]. Decreasing blood glucose and protective action on the kidney and pancreas injury of STZ diabetic rats [231]. Inhibition of hyperglycaemia-relevant α-glucosidase but not α-amylase [227, 232]. Antidiabetic activity might be due PPAR activation [233]. Possible renoprotective role in diabetic nephropathy [229].	Antioxidative [227, 234] action. Inhibited significantly the hypertension-relevant angiotensin I-converting enzyme [227]. Litholytic effects of herbal extracts on cystine urinary calculi [235]. Attenuating high-glucose-induced mesangial fibrosis and inflammation [229].
14	Polygonaceae *Rheum ribes* Linn. (Decoction of roots [23]) Tannins and hydroxyanthracene derivatives (rhein, physcion, aloe-emodin, chrysophanol, physcion-8-*O*-glucoside, aloe-emodin-8-*O*-glucoside, sennoside A, rhaponticin) [236, 237], minerals [238], phenolics (pyrocatechol) and flavonoids (quercetin equivalents) [239].	Insulin releasing effects in healthy mice [240] and hypoglycemic activity in alloxan-diabetic animals [241]. Significant dose dependent dual inhibition of α-amylase and α-glucosidase *in vitro*[48].	Antiviral [242] and antibacterial activities [243] with nutritional value [238]. Antioxidative potential [239, 244, 245]. Cytotoxic effects [246, 247] and anti-ulcer activity [248] as well as treating mild to moderate major depression disorders [249].
15	Rhamnaceae *Zizyphus spina- christi* (L.) Desf. (Infusion of fruits, leaves, bark [27]) Saponin glycosides [250–252]. Flavonoids [253, 254]. Essential oil [255, 256]. Amino acid, carbohydrate and lipid composition [257, 258].	Insulinotropic hypoglycaemic effects in diabetic rats [251, 259, 260]. Antidiabetic effect in alloxan-diabetic dogs [261].	Cytoprotective against liver aflatoxicosis [262, 263] and CCl_4_-fibrosis [264], vasoconstrictive effect in rat aorta [265]. Antiviral, antifungal and antibacterial activities [253, 266]. Its lipid fraction showed antimicrobial activity against *Bacillus subtilis*, *Escherichia coli* and *Streptococcus pyogenes*[257]. Its fruit and seed are good source of protein, mineral and energy foods [258]. Antinociceptive effect in mice and rats [267, 268]. Antidiarrhoeal benefits [269]. Mild dose dependent CNS depressant effect [270]. Molluscicidal property [94].
16	Rosaceae *Alchemilla vulgaris* L.(Decoction of leaves, roots [28]) Polyphenols [271–273], flavonoids [274–276], tannins [277], gallic acid [278].	Weight reduction in obese subjects [279] despite lack of antihyper-glycemic activity in STZ diabetes mice [280].	Antioxidative properties [271, 273, 275]. Mouth ulcers and wound-healing properties associated with pro-mitotic activity in epithelial cells and myofibroblasts [281, 282]. Activation of thyroid hormone synthesis [272]; antimicrobial with antiradical [277, 283, 284] as well as anxiolytic properties [285].
17	Rosaceae *Sarcopoterium spinosum* (L.) Spach. [Syn *Poterium spinosum* L.] (Infusion, decoction of roots [13, 24, 27–29]) Triterpenoids [286]. α-tocopherol [287], proanthocyanidines [288].	Traditionally used in the treatment of diabetes [289]. Hypoglycaemic effect, evidenced in rabbits, with fluctuations [290–292]. Antidiabetic properties viz. insulinotropic, and insulin sensitizing [293, 294]. Starch blocker due to duality of inhibition of α-amylase and α-glucosidase [48].	Action potential changes induced by its polyflavane on normal or hypoxic guinea pig myocardial strips [295]. Tumour inhibitory effects [296] and antioxidative properties [43]. Inhibited isoproterenol-induced lipolysis in 3T3-L1 adipocytes [293].
18	Umbelliferae *Ferula persica* Wild. (Decoction of roots and resin [23, 30]) Sesquiterpenes, persicasulphides A, B and C and umbelliprenin [297–303]. Several coumarins (farnesiferol A, B, badrakemone, gummosin) and a new coumarin, farnesiferone A) [303, 304]. Sesquiterpene coumarin glycosides [305, 306]. Essential oil [307–310].	Did not demonstrate any α-amylase inhibitory activity, thus lacking on significant hypoglycaemic effects in normoglycemic and STZ-hyperglycaemic rats [46].	Matrix metalloproteinases inhibition [297]. Umbelliprenin from *F. persica* roots inhibits the red pigment production in *Serratia marcescens*[299]. Antifungal activity [300]. Antioxidant, anti-inflammatory and lipoxygenase inhibitory properties and cancer preventive activity of umbelliferin [302, 303]. Farnesiferol A significantly inhibited the P-glycoprotein activity [305]. Antimicrobial effects [309]. Antigenotoxic activity via prevention of oxidative damage to DNA of rat lymphocytes [311] as well as cytotoxicity [312]. Umbelliprenin induced apoptosis in CLL cell lines [313].
19	Urticaceae *Urtica dioica* L. (Decoction of herb [26]) Polyphenolics [314–316]. Flavonoids [317–319]. Essential oil [320, 321]. Lignan glucosides [322]. Carotenoids [323].	Antidiabetic effect on high fructose fed rats [324]. Alpha-amylase inhibitory activity [325]. Antihyperglycemia in animal models via reduction of intestinal glucose absorption [326] and enhancement of insulin secretion by Langerhans Isletes [327] or inhibition of α-glucosidase [328]. Hypoglycemic and protective activities of β-cells of Langerhans in hyperglycemic rats [329]. Proliferation of the beta cells of the diabetic rats [330]. Chronic exposure (24 h) to *U. dioica* significantly enhanced glucose uptake in L6-GLUT4myc myoblast cells [331]. Anti-hyperglycemic effect in STZ-rats via potentiating insulin activity, thus enhancing glucose utilization [332] and plausible activation of the human peroxisome proliferator-activated receptor in glucose homeostasis [333]. Protective effect on hepatocytes of STZ rats [334], neuro-protective effect in diabetes-induced loss of pyramidal cells [335].	Antioxidant, antiradical, antimicrobial and antiulcerogenic effects [314–316, 336]. Antimicrobial activity [337]. Promotes learning performance in the brain of rats [338]. Immunostimulatory activity of the flavonoid fraction and intracellular killing activity of the isolated flavonoid glycosides suggesting that they could possibly be useful for treating patients suffering from neutrophil function deficiency and chronic granulomatous diseases [317]. Immunostimulatory activity [317, 318, 339]. Cardiovascular effects like hypotensive responses, through a vasorelaxing effect mediated by the release of endothelial NO and the opening of potassium channels, and through a negative inotropic action [340]. Beneficial for treatment of benign prostatic hyperplasia [341]. Platelet inhibitory activity [342]. Hepatoprotective in CCl_4_ treated rats [343] and protective effect on the liver in hepatic ischemia-reperfusion-injured rats [344]. Antifungal role [266]. Regulation of inflammatory gene expression [345]. Aromatase inhibitory activity [346].
20	Zygophyllaceae *Peganum harmala* Linn. (Decoction of seeds [30]) Flavonoid glycosides [347] and major β-carboline alkaloids (Harmaline, harmine, harmalol, harmol and tetrahydroharmine) [348–350].	Antidiabetic activity in C57BL/KsJ-db/db mice [351].	Antiplasmodial and vasorelaxant benefits [352]. Antileishmanial [353, 354], analgesic [355], anti-inflammatory [356], and antiplatelet activities [357]. Insecticidal activity [358–360], antibacterial, antifungal and antiviral propensities [361–366]. ACE-inhibitory activity [367, 368] and inhibition of human monoamine oxidase (MAO) [369]. *In vitro* cell-toxicity on cancerous cell-lines [370–372] as well as herbicidal activity [373].
